# The Hepatitis C Cascade of Care: Identifying Priorities to Improve Clinical Outcomes

**DOI:** 10.1371/journal.pone.0097317

**Published:** 2014-05-19

**Authors:** Benjamin P. Linas, Devra M. Barter, Jared A. Leff, Sabrina A. Assoumou, Joshua A. Salomon, Milton C. Weinstein, Arthur Y. Kim, Bruce R. Schackman

**Affiliations:** 1 HIV Epidemiology and Outcomes Research Unit, Section of Infectious Diseases, Boston Medical Center, Boston, Massachusetts, United States of America; 2 Department of Epidemiology, Boston University School of Public Health, Boston, Massachusetts, United States of America; 3 Department of Healthcare Policy and Research, Weill Cornell Medical College, New York, New York, United States of America; 4 Department of Global Health and Population, Harvard School of Public Health, Boston, Massachusetts, United States of America; 5 Department of Health Policy and Management, Harvard School of Public Health, Boston, Massachusetts, United States of America; 6 Massachusetts General Hospital Boston, Massachusetts, United States of America; University of Sydney, Australia

## Abstract

**Background:**

As highly effective hepatitis C virus (HCV) therapies emerge, data are needed to inform the development of interventions to improve HCV treatment rates. We used simulation modeling to estimate the impact of loss to follow-up on HCV treatment outcomes and to identify intervention strategies likely to provide good value for the resources invested in them.

**Methods:**

We used a Monte Carlo state-transition model to simulate a hypothetical cohort of chronically HCV-infected individuals recently screened positive for serum HCV antibody. We simulated four hypothetical intervention strategies (linkage to care; treatment initiation; integrated case management; peer navigator) to improve HCV treatment rates, varying efficacies and costs, and identified strategies that would most likely result in the best value for the resources required for implementation.

**Main measures:**

Sustained virologic responses (SVRs), life expectancy, quality-adjusted life expectancy (QALE), costs from health system and program implementation perspectives, and incremental cost-effectiveness ratios (ICERs).

**Results:**

We estimate that imperfect follow-up reduces the real-world effectiveness of HCV therapies by approximately 75%. In the base case, a modestly effective hypothetical peer navigator program maximized the number of SVRs and QALE, with an ICER compared to the next best intervention of $48,700/quality-adjusted life year. Hypothetical interventions that simultaneously addressed multiple points along the cascade provided better outcomes and more value for money than less costly interventions targeting single steps. The 5-year program cost of the hypothetical peer navigator intervention was $14.5 million per 10,000 newly diagnosed individuals.

**Conclusions:**

We estimate that imperfect follow-up during the HCV cascade of care greatly reduces the real-world effectiveness of HCV therapy. Our mathematical model shows that modestly effective interventions to improve follow-up would likely be cost-effective. Priority should be given to developing and evaluating interventions addressing multiple points along the cascade rather than options focusing solely on single points.

## Introduction

Recognizing that hepatitis C virus (HCV) is a highly prevalent but under-diagnosed infection, the U.S. Centers for Disease Control and Prevention (CDC) recently updated guidelines to recommend routine, one-time screening for HCV infection among all individuals born between 1945 and 1965 [1]. As these guidelines are implemented, the number of people with identified chronic HCV-infection will likely rise.

Nearly twenty years of experience with HIV treatment has led to a sophisticated understanding of the “cascade of care” that occurs between diagnosis and achieving durable HIV virologic suppression [2]. There is a similar cascade for HCV, which requires linking to HCV care, receiving confirmatory testing, staging disease, initiating therapy, and adhering to therapy despite adverse effects [3]. Compared to HIV, there are significant differences in benefits and costs of addressing the HCV cascade, because unlike HIV, effective HCV treatment results in a cure (sustained virologic response, SVR) [4], [5].

In the era of pegylated interferon and ribavirin-based HCV therapy, only 7–10% of those with identified HCV infection ever attained SVR [6]–[10]. As screening expands and treatments improve, there is growing interest in developing interventions to improve follow-up with HCV care after diagnosis [11]. Such interventions may target a single or multiple points along the HCV cascade of care, but there are no data to suggest which types of interventions along the cascade are likely to have the greatest impact on clinical or cost-effectiveness outcomes. For example, would limited resources be best employed to improve linkage to HCV care, or to improve the percentage of those already linked to care that initiates HCV therapy? Further, are resources best used to maximize follow-up at one point in the cascade where follow-up is particularly poor, or should we target multiple points simultaneously even if an intervention with multiple targets is somewhat less effective than a more narrowly targeted intervention at improving follow-up at any individual point?

Mathematical modeling provides a useful approach for comparing intervention strategies prior to intervention implementation and affords decision-makers with reasonable estimates as to whether the interventions, if effective, are likely to be the most efficient use of limited resources. Once priority strategies are identified through mathematical modeling, comparative effectiveness trials can be designed to test the efficacy of specific interventions, and implementation science can identify and address barriers to implementation [12].

We used the Hepatitis C Virus Cost Effectiveness (HEP-CE) model, a mathematical model of HCV disease progression and care delivery, to estimate the impact of loss to follow-up along the cascade of HCV care on clinical outcomes and costs, and to identify specific interventions that are promising candidates for future intervention design, evaluation, and implementation research. Each hypothetical intervention targeted one or more distinct points along the cascade of care, with different cost and implementation assumptions in order to identify the most effective and cost-effective strategies.

## Methods

### Overview

We used the Hepatitis C Cost-Effectiveness (HEP-CE) model, a Monte Carlo simulation of HCV natural history and care delivery, to simulate the progression of a cohort of HCV mono-infected individuals recently identified with HCV antibody (Ab) sero-reactivity. Details of the model are published elsewhere [13], [14] and are presented in Appendix S1. We sought to answer 3 questions:

1. How does loss to follow-up along the cascade of HCV care affect the clinical benefits of current and future therapies?
2. Which approaches to reducing loss to follow-up are likely to provide the best value for the resources invested and should therefore be prioritized for future development?
3. What are the likely program budgetary impacts and clinical outcomes of the simulated interventions?

Clinical outcomes included life expectancy and discounted quality-adjusted life expectancy (QALE). Process outcomes included the proportion of individuals linking to HCV care, initiating HCV therapy, and attaining SVR. The model also generated two cost estimates:

1. Mean discounted lifetime medical costs from a health system perspective - the costs of hospitalizations, emergency department visits, and outpatient visits, as well as the costs of interventions, and those of HCV treatment for the portion of patients who initiate HCV therapy. These health system perspective medical costs are discounted at 3% annually over a lifetime time horizon [15].
2. Program costs from the perspective of a program director implementing retention interventions – the costs of the intervention itself, undiscounted, over a 5-year time horizon.

### Model Structure

#### Cascade of care

**Figure 1:**
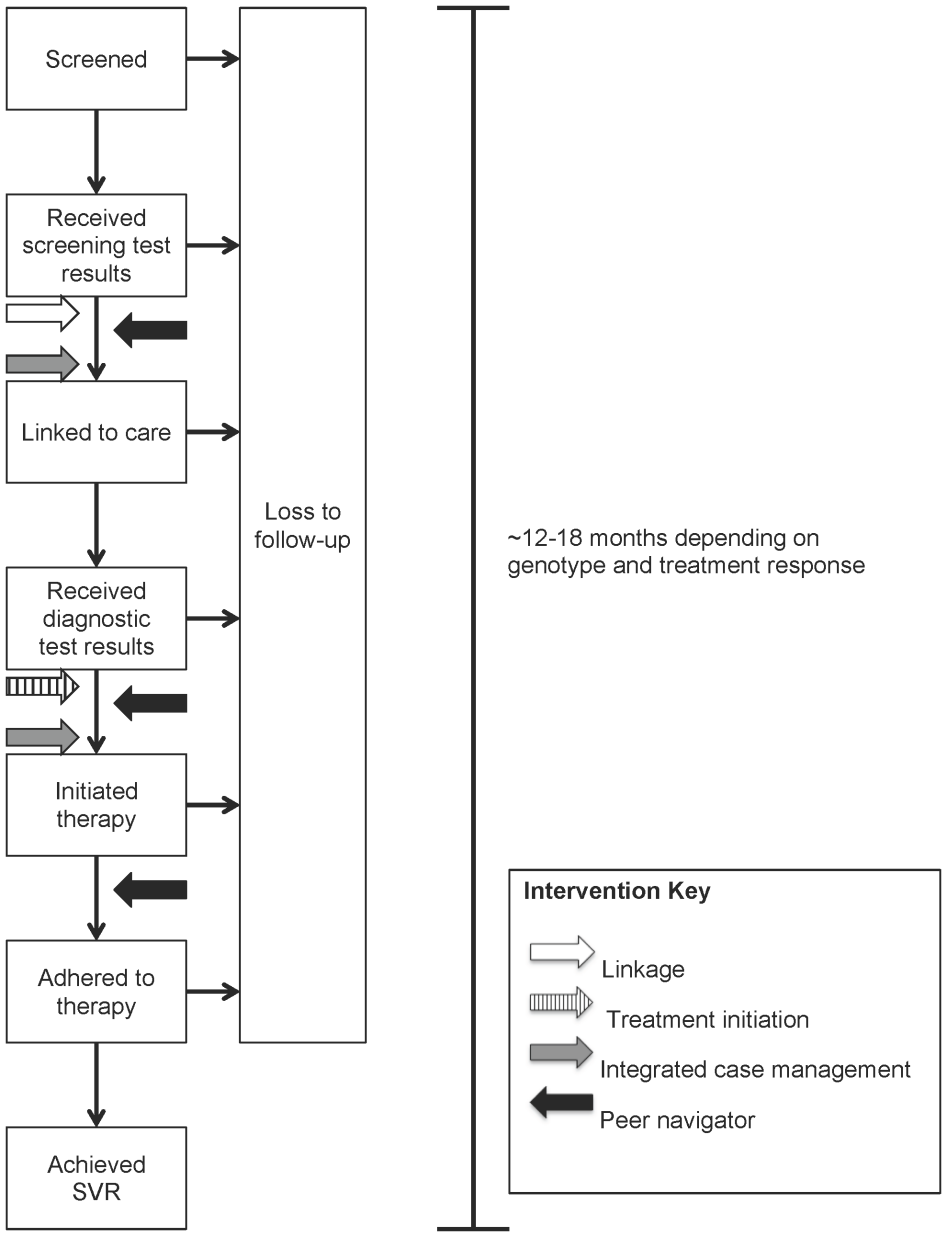
Cascade of care flow diagram. The flow diagram represents the steps of the HCV cascade of care, as well as key model parameters related to loss to follow-up. Arrows noted in the key represent points along the cascade at which candidate interventions improved follow-up. Individuals lost to follow-up prior to receiving their screening test results maintained a rate of re-screening such that their HCV status could be identified in the future (median time to first re-screen = 50 months). In addition, those who were lost to follow-up after obtaining screening test results had a monthly probability of re-linking to HCV care (median time to re-link = 32 months).

The simulated cohort includes chronically HCV-infected individuals who have been recently screened positive for HCV-infection. After screening, individuals enter a cascade of care in which they face a probability of completing each successive step, conditional upon having successfully navigated the previous step (Figure 1, Figure S1 in Appendix S1). We used estimates from published observational cohorts to inform the following parameter values:

1. Obtaining HCV screening results (74%) [16]–[18]
2. Linking to HCV care (53%) [16], [19]–[22]
3. Receiving diagnostic test results (98%) [7], [23]
4. Deciding on and initiating HCV therapy (27%) [8], [24], [25]
5. Adhering to and completing HCV therapy (83%–91%) [26]–[29]

When individuals fail to navigate a step in the cascade, they are considered lost to follow-up. Consistent with published data from observational cohorts, when patients are not engaged with HCV treatment their HCV disease progresses, they continue to experience decreased quality-of-life that is a function of their degree of fibrosis, and they continue to accrue health care costs related to their HCV-infection [14], [30]–[38]. Simulated individuals lost to follow-up maintain a probability of “re-linking” to care over the following 10 years as a result of re-testing or further engagement with the health care system.

#### HCV disease progression

Individuals with chronic HCV-infection progress in the model through 3 stages of liver disease: mild to moderate fibrosis, cirrhosis, and decompensated cirrhosis [39]. When histology reaches cirrhosis (median time 25 years from age of infection), individuals face a probability of mortality attributable to liver disease, from either complications due to cirrhosis or hepatocellular carcinoma [40]–[42]. Individuals develop cirrhosis at different rates. For example, some individuals begin the simulation with cirrhosis, while others never develop cirrhosis despite their HCV-infection. At all disease stages, HCV-infection is associated with increased health care costs and decreases in quality-of-life that were varied in sensitivity analyses [30]–[38].

Individuals who attain SVR are exposed to a risk of HCV re-infection [43]. Those who are re-infected may be re-treated, but only if they are screened for HCV and again navigate the HCV cascade of care. We assume that individuals who attain SVR are exposed to re-infection risk throughout the rest of their life, as on-going or relapsed injection drug use has been cited as a factor limiting the effectiveness of interventions to improve HCV treatment rates [44]. Such an assumption is conservative from the perspective of evaluating intervention efficacy, by reducing the impact of effective interventions. When re-infected, individuals resume HCV disease progression at the stage of fibrosis that they had reached during their prior HCV-infection.

#### HCV therapy

HCV treatment efficacy is a function of HCV genotype and fibrosis stage [26], [28], [29]. The base case HCV therapy regimens reflect the standard of care at the time that we completed the analysis. Individuals with genotype 1 infection receive 24–48 weeks of pegylated interferon (PEG), ribavirin (RBV), and telaprevir (TPV) combination therapy including early stopping criteria for treatment futility [45]. We chose TPV (rather than boceprevir) for the base case due to its straightforward treatment algorithm and because its higher upfront costs result in more conservative estimates of the cost-effectiveness of treatment [46]. In sensitivity analyses, we included a scenario that included the lower up-front cost of boceprevir. For those with genotypes 2 or 3 infection, we modeled PEG/RBV therapy [28], [29].

For all genotypes and regimens, in each month patients face a probability of withdrawal from therapy due to either treatment toxicity or non-adherence. Patients who withdraw from therapy due to toxicity accrue additional costs (Table S1 in Appendix S1). Patients who withdraw prior to the end of their intended treatment course stop accruing the costs of therapy and are not eligible to attain SVR; we did not include re-treatment in this analysis for those that fail.

Because HCV therapy is rapidly evolving, we also simulated a scenario in which individuals chronically infected with all HCV genotypes were treated with an oral, interferon-free regimen that avoids common toxicities associated with interferon [47], [48]. Because we anticipate that any specific interferon-free regimen could be replaced quickly by an even newer generation of therapy, we opted to simulate a hypothetical interferon-free option, rather than “over fit” the model to a specific treatment course. To that end, we modeled a 12-week course of oral interferon-free therapy for all HCV genotypes, without criteria for stopping therapy early for treatment futility. We used reports from phase 2 and 3 clinical trials of the nucleotide HCV polymerase inhibitor sofosbuvir to inform treatment efficacy [48]–[51]. We used the cost of a 12-week course of sofosbuvir and ribavirin as the cost of interferon-free therapy, and we varied this assumption widely in sensitivity analyses. We also assumed that individuals would be more likely to initiate interferon-free therapy compared to PEG/RBV regimens (54% vs 27%) and less likely to drop out of therapy because of reduced toxicity and improved convenience (Table 1).

**Table 1:** Model input parameters for a Monte Carlo simulation of HCV.

Variable	Base Case Value	Range Evaluated	Source
**Cohort characteristics**			
*** ***Mean age, years (S.D.)	55 (10)	45 (10) –65 (10)	[59]
*** ***Proportion male	0.63	0.40–0.80	[59]
*** ***Proportion with genotype 1	0.73	0.60–0.90	[75]–[78]
*** ***Average age at infection (years)	26	16–36	[60]
**Cascade of care variables**			
*** ***Proportion receiving screening test results	0.74	0.18–0.84	[16]–[18]
*** ***Proportion linking to HCV care a	0.53	0.45–0.93	[16], [19]–[22], See text
*** ***Proportion receiving diagnostic test results	0.98	0.95–1	[7], [23]
*** ***Proportion initiating HCV therapy b	0.27	0.19–0.67	[8], [24], [25], See text
*** ***10 year probability of re-engaging with care after being lost to follow-up	0.27	0–0.53	See text
**HCV disease progression**			
*** ***Median time from infection to cirrhosis (years)	25	10–40	[41], [42]
*** ***Median time from cirrhosis to first decompensation (years)	10.8	5.6–19.3	[40], [79]
*** ***Liver-related mortality with cirrhosis (deaths/100 PYs)	2.73	1.38–4.08	[80]
*** ***Incidence (infections/100 PYs)	0.66	0–1.32	[59]
*** ***Probability of clearing acute infection	0.26	0.22–0.29	[81], [82]
**HCV therapy efficacy**			
** ** *Genotype 1 (PEG/RBV/TPV)*			[26], [27]
*** ***Probability of withdrawal due to non-adherence	0.06	0.01–0.09	
*** ***Probability of withdrawal due to toxicity	0.11	0.01–0.16	
*** ***Probability of SVR for non-cirrhotics	0.75	0.60–0.95	
*** ***Probability of SVR for cirrhotics	0.63	0.60–0.95	
** ** *Genotype 2 or 3 (PEG/RBV)*			[28], [29]
*** ***Probability of withdrawal due to non-adherence	0.06	0.01–0.10	
*** ***Probability of withdrawal due to toxicity	0.03	0.01–0.06	
*** ***Probability of SVR for non-cirrhotics	0.74	0.55–0.95	
*** ***Probability of SVR for cirrhotics	0.58	0.55–0.95	
** ** *Interferon-free regimen*			[48]–[51].
*** ***Probability of withdrawal due to non-adherence	0.02	0–0.04	
*** ***Probability of withdrawal due to toxicity	0.006	0–0.010	
*** ***Probability of SVR for non-cirrhotics	0.90	0.80–1	
*** ***Probability of SVR for cirrhotics	0.81	0.70–0.90	
**Costs**			
*** ***Routine medical costs per month without HCV c	$140–$920	$70–$1,380	[83]
*** ***Routine medical costs per month with HCV c	$250–$1,500	$125–$2,250	[83], [84]
*** ***Diagnostic testing once screened positive d	$80	$40–$120	[54], [55]
** ** *Program costs per participant* e			
*** ***Linkage intervention	$1,883	$905–$4,518	[62]
*** ***Treatment initiation intervention	$1,021	$1,021–$4,475	[62], See text
*** ***ICM intervention	$2,191	$1,470–$6,716	[62], See text
*** ***Peer navigator intervention	$5,344	$1,243–$5,344	[11], [62]
** ** *HCV therapy costs per month*			
*** ***Provider visit costs f	$121	$61–$182	[54], [55]
*** ***PEG g	$1,572–$2,097	$786–$3,146	[46]
*** ***RBV h	$685–$1,371	$343–$2,057	[46]
*** ***TPV i	$15,154	$7,577–$22,731	[46]
*** ***Filgrastim j	$1,900	$950–$2,850	[46]
*** ***Clobetasol propionate k	$160	$80–$240	[46]
**Complete course genotype 1** l	$67,530–$89,742	$44,871–$134,613	[46], [55]
**Complete course genotype 2/3**	$22,627	$11,314–$33,941	[46], [55]
**Complete course of IFN-free (all genotypes)**	$91,500	$80,000–$200,000	See text
*** ***Managing treatment ending toxicity	$361	$181–$542	[46], [52], [53], [55]
**Quality of life**			
*** ***Without HCV infection m	0.90	0.80–1.0	[85]–[87]
*** ***HCV with no to moderate fibrosis	0.89	0.75–1.0	[32], [34], [37]
*** ***HCV with cirrhosis	0.62	0.55–0.75	[32], [34], [37]
*** ***HCV after first decompensation event	0.48	0.40–0.60	[32], [34], [37]
*** ***On HCV treatment n	0.90	0.84–0.96	[88]
*** ***Major toxicity decrement o	0.16	0.09–0.25	[89]

S.D. = standard deviation; PY = person-year; PEG = pegylated interferon; RBV = ribavirin; TPV = telaprevir; SVR = sustained virologic response; ICM = integrated case management; IFN = interferon.

a The lifetime probability of linking to HCV care upon receipt of a positive antibody result is 66%.

b In the interferon-free scenario, we assumed that 54% of those linked to care would initiate therapy.

c Costs varied as a function of age and sex.

d Includes the cost of a RNA confirmatory test and a nursing visit.

e ntervention costs are presented on a per participant basis, assuming that the participant completes the entire intervention. During the simulation, participants accrued costs on a monthly basis. If the participant was lost to follow-up, or otherwise withdrew from care before the end of the intervention, then that patient stopped accruing intervention costs at the time of being lost (see Appendix S1 for details).

f Treatment visit costs are higher in the first month compared to other months.

g 13% of patients received a reduced weekly dose of 135 mcg in response to non-treatment ending neutropenia [45].

h RBV dose was a function of genotype (genotype 1 = 1,200 mg/day; genotype 2 or 3 = 800 mg/day). In addition, 36% of patients on triple therapy and 17% on dual therapy were treated with reduced dose RBV = 600 mg/day in response to non-treatment ending anemia [45].

i Only patients with genotype 1 receive TPV for treatment months 1–3.

j 13% of patients developed non-treatment ending neutropenia (absolute neutrophil count <750/ml) and received filgrastim 300 mcg/two times weekly [45].

k Only patients with genotype 1 treated with PEG/RBV/TPV therapy received 150g/month for treating mild rash (28% during the first 3 months of therapy) [45].

l The range reflects the fact that some patients were treated for 6 months, while those without rapid virologic response were treated for 12 months.

m Reflects lower quality of life for individuals with HCV risk-factors such as substance use.

n This utility weight was multiplied by an individual’s health state utility during the months that a patient was receiving HCV therapy without major toxicity. For example, a patient with HCV and mild to moderate fibrosis who underwent HCV treatment had a utility = 0.801 (0.90×0.89) during the months that (s)he was on medications.

o This utility “toll” was subtracted from a patient’s health state utility during the month of a major toxicity event.

Treatment costs include those of medications, provider visits, laboratory monitoring, and management of common toxicities (Table 1, Table S2 in Appendix S1) [46], [52]–[55]. Consistent with findings from large cohort studies, successful HCV therapy results in cessation of HCV-related disease progression, reduction in liver-related mortality and health care resource utilization, and return of quality-of-life to that of age-matched HCV-uninfected individuals [4], [56]–[58].

### Analyses

#### Cohort

The simulated cohort was comprised of one million chronically HCV-infected individuals whose demographic and clinical composition matched those of HCV-infected individuals in the U.S. [59]. The mean age was 55 (standard deviation 10) years and the cohort was 63% male [59]. Reflecting the cohort age, early age at infection (26 years), and median time to developing cirrhosis (25 years from age of infection), 46% of the cohort had cirrhosis at simulation baseline (Table 1) [41], [60]. We excluded individuals co-infected with HIV because these individuals have different opportunities for cascade of care interventions and different HCV treatment outcomes.

#### Impact of imperfect follow-up on clinical outcomes

We used the model to simulate the cohort under 2 scenarios. First, we assumed status quo rates of loss to follow-up along each point in the cascade of care. Second, we assumed an optimal scenario in which medical contraindications to interferon and medication-related toxicity continued to limit HCV treatment initiation and completion, but in which follow-up along the cascade was perfect and, in patients who did not have drug-related toxicity, adherence to therapy was perfect. In the optimal follow-up scenario, 100% of those identified as HCV-infected linked to care, those without a medical contraindication initiated therapy (55%), and the only reason for withdrawal from HCV therapy was medication-related toxicity [8], [24], [25], [61]. We attributed the difference in outcomes between the 2 scenarios to the loss to follow-up along the cascade.

#### Simulated interventions to improve follow-up

We modeled 4 hypothetical interventions to improve HCV outcomes:

1. Linkage intervention - a 3-month intervention based on the Anti-Retroviral Treatment and Access to Services (ARTAS) case management program, which includes up to 5 visits with a case manager and is designed to improve linkage to care rates, at a cost of $1,900/patient [62], [63].
2. Treatment initiation intervention - a 3-month intervention targeting individuals already engaged in HCV care to enhance the probability of initiating treatment prior to treatment start. We used expert opinion to describe a hypothetical intervention that includes an extended visit with a physician, 2 nursing visits, and supportive services from a case manager (such as assistance with insurance forms, obtaining public benefits, and coordinating appointments) at a cost of $1,000/patient.
3. Integrated case management (ICM) intervention - a 6-month intervention that uses case managers to improve both linkage to HCV care as well as the probability of initiating treatment at a cost of $2,200/patient. ICM combines the components of both the linkage and treatment initiation interventions and is designed to occur before treatment start.
4. Peer navigator intervention – a 12–18-month intervention that uses peer navigators to work with clients from the time they are diagnosed as HCV-infected through the completion of HCV treatment. We modeled the peer navigator intervention on the New York City Department of Public Health and Mental Hygiene’s “Check Hep C” program [11]. The intervention encompasses the 3–6 month period patients spend in HCV care prior to starting therapy through the 6–12 months (depending on HCV genotype and response to treatment) patients spend on HCV treatment at a cost of $5,300/patient.

#### Intervention effectiveness

The interventions affected one or more point(s) along the HCV cascade of care (Figure 1). We modeled the effectiveness of the linkage intervention by increasing the probability that an individual with chronic HCV-infection with recently identified reactive HCV serum Ab would present to HCV care for evaluation. For the treatment initiation intervention, we increased the proportion initiating HCV treatment after linking to care. For the ICM intervention, we increased both the probability of linking to HCV care and the probability of initiating HCV therapy. Finally, we modeled the peer navigator intervention by increasing the probability of linking to HCV care and initiating treatment, and decreasing the rate of withdrawal from HCV therapy due to non-adherence.

We used expert opinion to develop a base case effect size for a successful intervention strategy. We assumed that effective interventions would increase follow-up at each targeted point(s) along the cascade of care by 10 absolute percentage points. We varied this assumption in sensitivity analyses from 2 to 40 percentage point absolute increases in the effectiveness of the interventions at each of their targeted points in the cascade. In further sensitivity analyses we altered the approach to model a 10% *relative* improvement in follow-up at the relevant points along the cascade. Additionally, we conducted analyses in which we assumed that interventions that simultaneously target multiple points along the cascade have less impact at any single point compared to interventions that target a single point.

The interferon-free treatment scenario included several key changes to both the standard of care and interventions strategies including:

1. Higher probability that individuals would initiate HCV therapy in the absence of an intervention, reflecting the improved tolerability of an IFN-free regimen.
2. Lower cost for the peer navigators, reflecting the shorter treatment duration using IFN-free therapy.
3. Lower probability of non-adherence in the absence of a peer navigator, reflecting the elimination of weekly interferon injections and a lower toxicity profile.

#### Program costs

Program costs included labor, materials, and overhead related to administering a hypothetical intervention. We used public health literature, U.S. Bureau of Labor Statistics data and Medicare reimbursement schedules to identify the materials and human resource costs needed to implement the hypothetical interventions (Table 1, Table S3 and Table S4 in Appendix S1) [11], [62]–[66].

The model applies program costs on a monthly basis only during months in which an individual receives care; if an individual is lost to follow-up before completing an intervention, the subsequent monthly program costs are not incurred. The model sums the accrued undiscounted total program costs over a five-year time horizon. To estimate lower and higher cost scenarios for each intervention, we varied the average caseload that intervention staff members could carry.

#### Incremental cost-effectiveness

We calculated the incremental cost-effectiveness ratio (ICERs) of an intervention compared to the next best alternative as the additional cost divided by the QALY gain ($/QALY) [67], [68]. Interventions that had higher costs but fewer QALYs gained, as well as those that had a higher cost per QALY than a more effective intervention were considered “dominated” and no ICER was calculated. All costs are in 2011 U.S. dollars. QALYs and costs for ICERs were discounted at 3% annually [68]. For purposes of interpreting cost-effectiveness analyses, we assumed a U.S. societal willingness to pay threshold of $100,000/QALY gained [69], [70].

## Results

### Outcomes of Imperfect Follow-up

**Figure 2:**
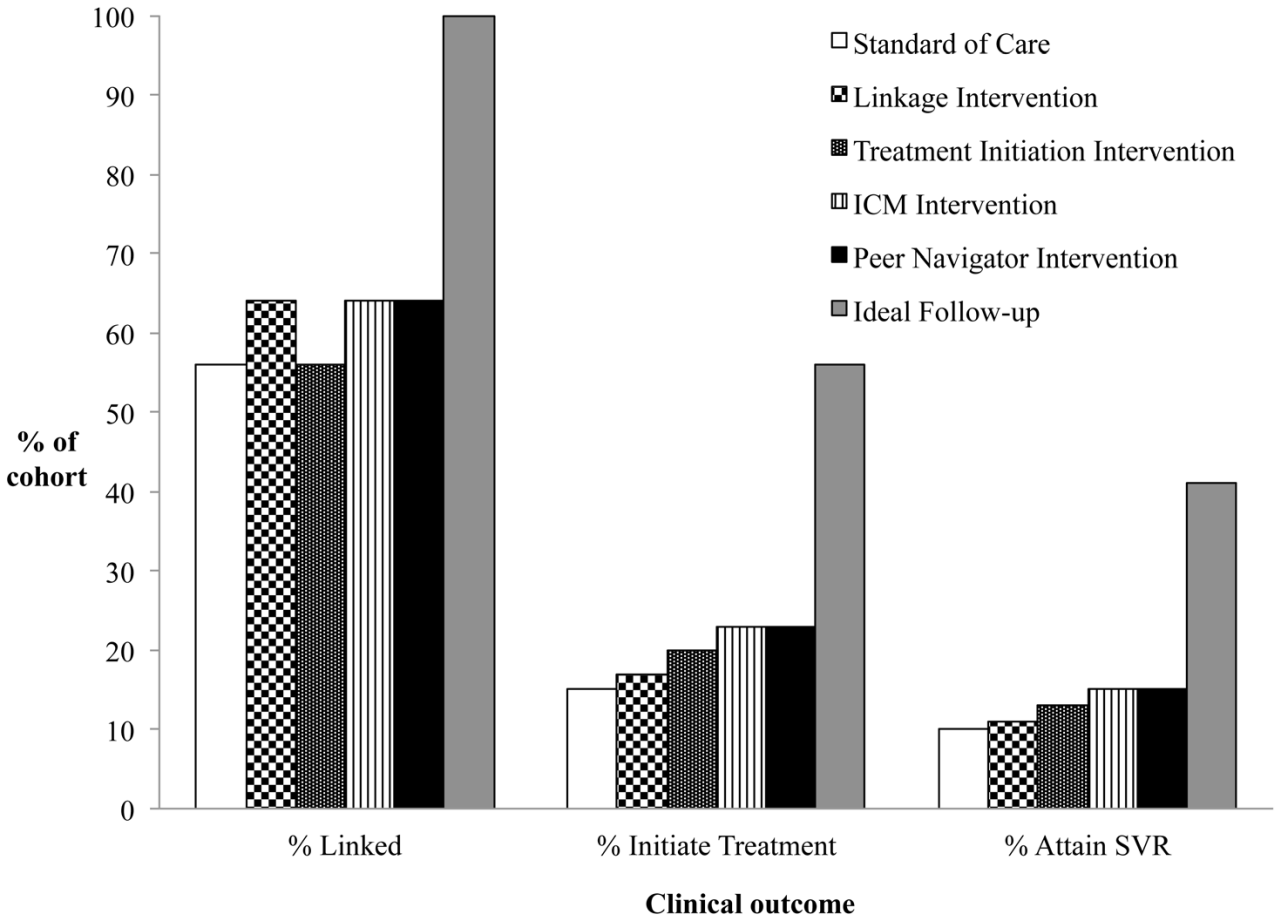
Intervention clinical outcomes. The bar graph illustrates the percent of the cohort attaining clinical outcomes along the HCV cascade of care. Each bar shading represents a specific intervention scenario.

When we assumed the current standard of care (SOC), we estimated that 15% ever initiated HCV treatment, and 10% ultimately attained SVR (Figure 2). When we assumed ideal follow-up along the cascade of care, we estimated that 56% ever initiated HCV treatment and 41% attained SVR. Thus, due to loss to follow-up, we estimate that the proportion achieving SVR was approximately 25% of the theoretical best-case scenario.

### Interventions to Improve Follow-up

Simulating a hypothetical intervention that improved linkage to care from 53% to 63% resulted in a 14% increase in the number attaining SVR compared to the current SOC (Figure 2). Mean life expectancy increased from 21.30 to 21.36 years, QALE from 9.99 to 10.06 QALYs, and discounted lifetime medical costs from $189,000 to $190,700 (Table 2).

**Table 2:** Projected incremental cost effectiveness ratios of potential interventions to improve HCV follow-up.

Strategy	Undiscounted	Discounted		Incremental		ICER ($/QALY)
	Life Expectancy	Cost ($)	QALY	Cost ($)	QALY	
**Standard of Care**	21.30	189,000	9.99	–	–	–
**Linkage**	21.36	190,700	10.06	1,700	0.07	dominated a
**Treatment Initiation**	21.50	193,100	10.21	2,400	0.15	dominated b
**Integrated Case Management**	21.59	194,800	10.30	1,700	0.09	18,900
**Peer Navigator**	21.60	195,300	10.31	500	0.01	48,700 c

QALY = Quality-adjusted life year; ICER = incremental cost-effectiveness ratio.

Costs and QALYs are lifetime and discounted at an annual rate of 3%. Costs are in 2011 U.S.$ and rounded to the nearest $100. All QALYs are rounded to the nearest hundredth.

a The ICER of linkage compared to standard of care is $26,500/QALY gained; linkage is extended dominated.

b The ICER of treatment initiation compared to standard of care is $19,200/QALY gained; treatment initiation is extended dominated.

c The ICER of peer navigators compared to standard of care is $20,000/QALY gained.

When we simulated a similarly effective intervention that improved treatment initiation from 27% to 37%, we observed an 18% increase in the number attaining SVR compared to the linkage intervention and an estimated 36% increase compared to SOC. Life expectancy was 21.50 years, greater than that of both the SOC and the linkage intervention scenarios. QALE was 10.21 QALYs, and discounted lifetime medical costs were $193,100. When considering only the 2 hypothetical interventions that intervened at a single point along the cascade, the more distally targeted intervention along the cascade (treatment initiation) dominated the more proximally targeted intervention (linkage), meaning that it provided longer life expectancy than linkage at a lower cost per QALY gained.

A hypothetical ICM program that improved both linkage and treatment initiation by 10 percentage points resulted in the same number of patients linking to care as the linkage intervention, but it resulted in more patients initiating therapy and attaining SVR than either the linkage or treatment initiation interventions alone. As a result, we found that the ICM intervention dominated both linkage and treatment initiation interventions with an ICER compared to SOC of $19,100/QALY gained (Table 2).

Finally, we estimated that implementing a hypothetical comprehensive peer navigator program would provide a 1.0% increase in the proportion of patients achieving SVR compared to the ICM program. We found that the hypothetical peer program extended life expectancy, QALE, and costs compared to ICM with an ICER of $48,700/QALY gained.

### Program Costs

For a cohort of 10,000 hypothetical HCV-infected individuals, the undiscounted 5-year cost of implementing each simulated intervention was $6.4 million for linkage, $7.6 million for treatment initiation, $11.5 million for ICM, and $14.5 million for peer navigators.

### Sensitivity Analyses on Intervention Effectiveness and Costs

**Figure 3:**
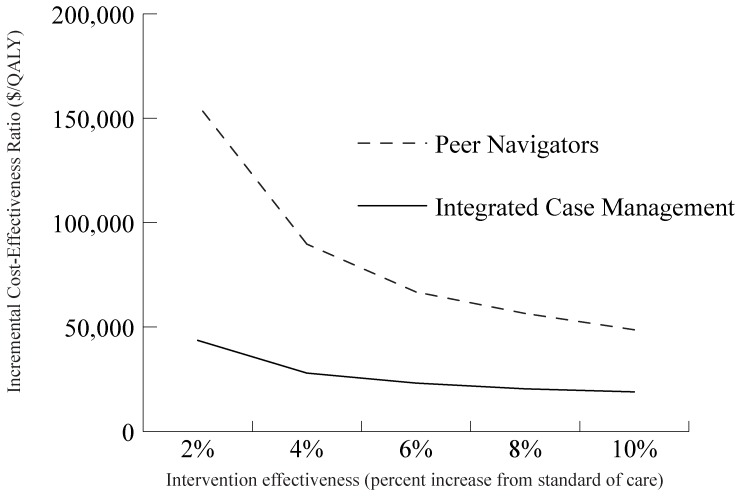
Incremental cost-effectiveness ratios (ICERs) of increased intervention effectiveness. The line graph illustrates the incremental cost-effective ratio (ICER) of the peer navigator and integrated case management hypothetical interventions compared to the next best alternative across a range of intervention effectiveness.

The projected ICERs for the simulated ICM and peer navigator interventions remained <$100,000/QALY across broad assumptions about intervention effectiveness (Figure 3). The ICER of ICM remained <$50,000/QALY gained, even when we assumed that all interventions improved follow-up by only 2 percentage points. Similarly, we found that the ICER of peer navigators relative to ICM remained <$100,000/QALY gained unless all interventions improved follow-up by fewer than 4 percentage points.

The ICER of peer navigators compared to ICM was sensitive to the effectiveness of the peers. With any assumption of decreased retention relative to ICM, peer navigators no longer had an ICER <$100,000/QALY gained, even when we assumed that the labor cost of the peers was lower than that of case managers.

When we reduced the effectiveness of both the simulated peer navigator and ICM interventions compared to interventions that solely targeted linkage or treatment initiation, peer navigators continued to be more effective and provided the best value for money. We estimated that only when the outcomes of peer navigators and ICM were less than 80% of the treatment initiation intervention did the treatment initiation intervention become preferred.

When we modeled intervention effectiveness as a 10% *relative* improvement (rather than a 10 percentage point absolute improvement) compared to SOC, results were similar. Again, when considering only single point interventions, the more distally targeted intervention along the cascade of care (treatment initiation) dominated the proximally targeted intervention (linkage). Both single point interventions, however, were economically inefficient compared to hypothetical interventions that targeted multiple points along the cascade (ICM and peer navigators). Peer navigators remained the preferred strategy with an ICER of $35,900/QALY gained compared to ICM.

When we increased the cost of ICM, the ICER of ICM compared to its next best alternative remained <$100,000/QALY gained as long as the estimated cost of ICM was less than $2,900 per participant (base case $2,191). We found that at higher costs, ICM was no longer efficient as peer navigators provided a greater life expectancy benefit at lower cost per QALY gained. Likewise, the ICER of the peer navigators was less than $100,000/QALY as long as the cost of the intervention was less than an estimated $6,700 per participant (base case $5,344).

### Sensitivity Analyses Assuming Interferon-free Therapy

With the availability of interferon-free therapy, assuming the same linkage rates, but an improvement in treatment initiation and adherence to therapy compared to current therapy, we estimated that 27% of individuals attained SVR. Life expectancy increased from 21.30 to 22.08 undiscounted life years, and QALE increased from 9.99 to 10.87 QALYs. With interferon-free treatment, peer navigators dominated all other interventions by providing additional SVR benefits at a lower cost per QALY gained with an ICER of $16,200/QALY gained compared to SOC. Life expectancy with peer navigators was 22.49 years, QALE was 11.32 QALY, and discounted, lifetime medical costs were $207,300. When we assumed that adherence to IFN-free therapy would be lower in the real-world than it was in clinical trials, peer navigators continued to dominate all other interventions.

### Additional Sensitivity Analyses

When we varied other model parameters including cohort characteristics, HCV disease progression, HCV therapy efficacy, costs and quality-of-life, all of the simulated interventions had ICERs <$100,000/QALY gained compared to the SOC, and linkage and treatment initiation interventions were consistently dominated by either ICM or peer navigators. When we assumed a longer median time from infection to the development of cirrhosis (40 years), which corresponded to a lower prevalence of cirrhosis at simulation baseline (18%), ICM and peer navigators continued to dominate linkage and treatment initiation interventions, and the ICER of peers compared to ICM was $51,200/QALY. All hypothetical interventions became more economically attractive (lower ICERs) when we assumed greater treatment efficacy and increased HCV-attributable morbidity and mortality. When we assumed less withdrawal from therapy due to non-adherence, the ICER of peer navigators compared to ICM increased substantially, and ICM was the preferred intervention. Assumptions about the costs of HCV medications and management of HCV treatment had little impact on findings.

## Discussion

Using mathematical modeling, this analysis estimates that loss to follow-up along the cascade of HCV care reduces the effectiveness of current HCV therapy by approximately 75%. We found that without improvement in loss to follow-up along the HCV cascade of care, the proportion of chronically HCV-infected individuals who achieve SVR will likely not change substantially from approximately 10%. More tolerable and effective interferon-free therapy will likely improve outcomes, but even assuming improved efficacy and a doubling in the proportion of patients initiating HCV treatment, we project that only 23% of individuals identified with chronic HCV-infection would be cured.

Investments in interventions to improve linkage to care, treatment initiation, and adherence to HCV therapy are needed. Our findings suggest that these potential interventions are likely to have attractive cost-effectiveness ratios when compared to the current SOC. Our work also demonstrates that interventions addressing multiple points along the cascade, including distally targeted points such as treatment initiation and therapy adherence, will likely provide better outcomes at more attractive ICERs than those targeting either a single point, or targeting points at the proximal end of that cascade, such as linkage.

There are two reasons that comprehensive interventions may be preferred to a targeted approach: first, interventions that address distally targeted points in the cascade have a greater impact on clinical outcomes than those that address loss to follow-up at earlier phases. The finding that distally targeted points in the cascade are critical is not unique to HCV-infection, as similar findings have been reported for conditions such as hypertension and HIV [71], [72]. Second, because the number of people who reach the end of the cascade of HCV care is a multiplicative function of the probability of loss to follow-up at every point along the cascade, interventions that improve follow-up at multiple points create a synergy of effects that may justify the greater resources required. For example, we found that a hypothetical peer navigator intervention was preferred to a hypothetical treatment initiation intervention unless the cost was over six times that of a treatment initiation intervention. Additionally, based on our assumptions, comprehensive interventions are more effective than targeted interventions even when their impact at any single step in the cascade of care is reduced by one fifth compared to an intervention that devotes all of its resources to improvement at a single step. Our results suggest that future studies should prioritize the development and evaluation of comprehensive interventions such as peer navigators or integrated case management, as these interventions are likely to provide not only better outcomes than linkage or treatment initiation interventions, but also better value for the resources invested.

There are limitations to this analysis. First, this is a simulation modeling analysis that relies on projections of the effectiveness and costs of hypothetical interventions. The simulation approach, however, provides guidance needed to inform and prioritize potential efforts to improve HCV care. The goal of this analysis is not to report the cost-effectiveness of a real-world program. Rather, we seek to simulate outcomes with hypothetical interventions in order to develop priorities for prospective, hypothesis-driven evaluation. We carefully considered all of the components of interventions, including overhead and administrative costs, using existing HIV and HCV interventions as models. In sensitivity analyses, we considered a variety of scenarios varying effectiveness and cost. The finding that comprehensive approaches are more economically attractive than single-point interventions was consistent across the range of reasonable assumptions.

Additionally, while we considered a variety of strategies to increase the number of people navigating the HCV cascade of care, we did not model alternative approaches to HCV treatment itself. For example, we did not model strategies that use IL28B genotyping to prioritize patients for protease-based therapy. Previous work indicates that such an approach may be cost-effective [73], [74]. Our goal in this analysis, however, was to focus on the cascade of care itself, not to investigate the cost-effectiveness of the accepted standard of HCV therapy. Were we to model both interventions to improve follow-up along the cascade, and novel treatment strategies, the relative contributions of multiple simultaneous interventions would be difficult to interpret. Any treatment algorithm that improves the value of HCV therapy in terms of cost per QALY gained, however, will also improve the value of interventions that increase the number of people starting therapy. As a result, novel approaches that improve the economic value of HCV therapy will likely improve the cost-effectiveness of cascade of care interventions and our results remain conservative.

Third, we included costs from a health system perspective, and therefore did not include patient time in the analysis. Relative to HCV treatment and intervention costs, patient time is a small percentage of total cascade of care costs. We varied intervention costs widely, and these sensitivity analyses may be interpreted as scenarios with and without patient time costs.

Finally, the base case analysis assumes HCV treatment using an HCV protease inhibitor in combination with interferon, which will not be the standard of care in the future. Given the rapid pace of HCV drug discovery, a modeling approach is advantageous as it projects costs and effectiveness under a variety of assumptions about future treatment. We considered a scenario utilizing more effective and less toxic interferon-free therapy based on available data, and we projected that comprehensive interventions such as peer navigators are more economically attractive as therapy becomes more costly and effective, even at our assumed increased cost of interferon-free therapy.

In conclusion, this analysis demonstrates that although nearly any effective intervention to improve follow-up in the HCV cascade of care will likely improve HCV outcomes, comprehensive approaches that focus on multiple points along the HCV cascade, such as peer navigators or integrated case management, may provide the best value for money and should be prioritized for future development and prospective evaluation.

## Supporting Information

Appendix S1 Appendix with supporting information, figure, and tables.
(DOCX)
