# Supplementary material for: The Hepatitis C Cascade of Care: Identifying Priorities to Improve Clinical Outcomes
Source: PLoS One. 2014 May 19;9(5):e97317. doi: 10.1371/journal.pone.0097317 (PMC4026319; doi:10.1371/journal.pone.0097317)
Supplement: Appendix S1 — Appendix with supporting information, figure, and tables. (DOCX) [file pone.0097317.s001.docx]

**Supporting Information**

**The HCV Cascade of Care: Identifying Priorities to Improve Clinical Outcomes**

Benjamin P. Linas^1,2^

Devra M. Barter^1^

Jared A. Leff^3^

Sabrina A. Assoumou^1^

Joshua A. Salomon^4^

Milton C. Weinstein^5^

Arthur Y. Kim^6^

Bruce R. Schackman^3^

^1^HIV Epidemiology and Outcomes Research Unit, Section of Infectious Diseases, Boston Medical Center, Boston, MA, United States of America; ^2^Department of Epidemiology, Boston University School of Public Health, Boston, MA, United States of America; ^3^Department of Healthcare Policy and Research, Weill Cornell Medical College, New York, NY, United States of America; ^4^Department of Global Health and Population, Harvard School of Public Health, Boston, MA, United States of America; ^5^Department of Health Policy and Management, Massachusetts General Hospital Boston, MA, United States of America; ^6^Harvard Medical School, Boston, MA, United States of America

Address correspondence to:

Benjamin P. Linas, MD, MPH

Boston Medical Center

HIV Epidemiology and Outcomes Research Unit

850 Harrison Ave

Dowling- 3N Room 3205

Boston, MA 02118

[Benjamin.Linas@BMC.org](mailto:Benjamin.Linas@BMC.org)

617-414-5238 (Voice)

617-414-7062 (Fax)

Portions of this paper were presented at the 20th Conference on Retroviruses and Opportunistic Infections and Prevention in Atlanta, GA in March 2013. This work has not been published, nor is it being considered by any other journal.

**Appendix S1**

**INTRODUCTION**

The analyses reported in the main manuscript utilize the Hepatitis C Cost-Effectiveness (HEP-CE) model of screening, care, and treatment for chronic (or acute) Hepatitis C Virus (HCV) infection. HEP-CE is a detailed simulation of HCV epidemiology, screening, linkage to care, treatment, and outcomes. The HEP-CE model tracks clinical outcomes including number of detected HCV antibodies, number linked to HCV care, number of cases of chronic HCV identified, number of HCV-infected people initiating therapy, number achieving sustained viral response (SVR), HCV-related mortality, quality of life, undiscounted life expectancy, and discounted quality adjusted life expectancy (QALE). In addition, the model generates estimates of discounted, lifetime medical costs from the health system perspective, as well as non-discounted program costs for interventions to improve HCV follow-up from the payer perspective. This appendix provides details of the model functionality, with explanations as to how we utilized the specific model features for this analysis. In addition, we provide a figure and several tables detailing input parameter values.

**HEP-CE MODEL**

The HEP-CE model is a Monte Carlo simulation of HCV-infection designed to estimate the outcomes and costs associated with various strategies of screening, treatment, and improving HCV care. The model includes 5 components: 1) HCV epidemiology, 2) HCV screening and linkage to care, 3) HCV disease progression, 4) HCV therapy, and 5) Non-HCV mortality.

HCV epidemiology

The incidence of HCV is a model input (rather than a model output as it is in some epidemiologic modeling exercises). In every month, individuals in the model who are not HCV-infected face a risk of acquiring HCV that is a function of their age and HCV risk behaviors (typically interpreted as current injection drug use versus no current use). Once HCV-infected, individuals begin the simulation of HCV disease progression (see below). For the purpose of this analysis, which simulates the progression of a group of chronically HCV-infected individuals who were recently screened for HCV-infection, all individuals were HCV-infected at the start of the simulation. Those who attained sustained virologic response (SVR), however, were no longer infected and were exposed to a risk of reinfection.

HCV disease progression

Incident cases of HCV-infection begin in the acute HCV health state. A proportion of individuals develop symptoms of acute hepatitis and are identified as acutely infected based on symptoms. Some of those with acute HCV-infection also experience spontaneous resolution of their infection, in which case they revert back to the HCV uninfected health state. Those not identified nor successfully treated for HCV, and whose infection does not spontaneously resolve, move to the chronic HCV infection health state. For the purpose of this analysis, all hypothetical individuals had chronic HCV-infection at simulation baseline. Those who attained SVR and were subsequently re-infected entered the acute HCV health state as part of the simulation of disease progression of their second infection event.

Chronic HCV is characterized in the model by 3 stages of linear fibrosis progression including mild-to-moderate fibrosis, cirrhosis, and decompensated cirrhosis. At all stages of fibrosis, HCV-infection is associated with increased costs and decreased quality of life. Mortality attributable to HCV-infection, however, does not accrue until an individual reaches his/her unique time of developing cirrhosis. Once an individual reaches the cirrhosis stage, (s)he faces a probability of liver-related mortality that remains constant unless s(he) initiates treatment and attains SVR.

To estimate critical disease progression event times, including the time at which an individual develops cirrhosis (t_cirrhosis_) and decompensated cirrhosis (t_decomp_), the model uses a stochastic process to draw 4 critical parameter values: 1) age at HCV-infection (Age_HCV_); 2) age at simulation base line (Age_base_); 3) months from infection to cirrhosis (m_cirrhosis_); and 4) months from cirrhosis to first liver decompensation event (m_decomp_).

For those with prevalent HCV infection at simulation baseline:

t_cirrhosis_  _=_ m_cirrhosis_ - (Age_base_ - Age_HCV_)

t_decomp_ _=_ t_cirrhosis_  _+_ m_decomp_

For those who contract incident HCV during the simulation:

t_cirrhosis_  _=_ m_cirrhosis_

t_decomp_ _=_ t_cirrhosis +_ m_decomp_

As a result of the stochastic process that defines times to cirrhosis and decompensation, not all HCV-infected patients in the model develop cirrhosis, and not all patients with cirrhosis die of HCV-attributable causes.

With spontaneous clearance or successful HCV treatment, disease progression ceases, and quality of life, costs, and mortality risk return to that of HCV-uninfected individuals of the same age, sex, and HCV risk behaviors. Re-infected individuals resume the course of fibrosis progression at the stage reached during prior infection.

HCV screening and linkage to care

The model provides flexibility to define various strategies for screening based on different screening modalities and intervals. All screening tests are characterized by a cost, sensitivity, and specificity. Test characteristics are a function of time since infection. The model distinguishes between an individual’s true infection status and clinical awareness of infection. Only individuals with identified HCV infection are eligible for HCV therapy.

The user can define a monthly probability that individuals present to an HCV screening site, as well as risk group-stratified probability that an individual is offered an HCV screening test. Subsequently, individuals offered a screening test have the probability of accepting or rejecting the offer. Those screened face a probability of not returning to receive their test results. Individuals who are screened as positive and receive their test result then have a user-specified probability of linking to HCV specialist care, obtaining confirmatory testing results with associated additional costs, and initiating and adhering to HCV therapy. Individuals can be lost to follow-up at each stage in this cascade of care. When lost, individuals continue with HCV- infection in the model, but are not eligible for HCV treatment unless they reengage with HCV care in the future. If individuals re-engage with HCV care, they are assumed to link to an HCV care provider, but they maintain a user-specified probability of initiating therapy.

HCV therapy

The model provides flexibility to simulate a variety of algorithms for HCV therapy, including distinct treatment protocols for acute and chronic HCV-infection, as well as for genotype 1 vs. genotype 2/3 infection. Treatment efficacy is modeled using 6 key parameters, each of which is stratified by cirrhosis status:

1. Probability of developing HCV RNA >1,000 copies/ml = P_failure_
2. Probability of attaining HCV RNA < assay at treatment week W_4_ = P_RVR_
3. Probability of attaining HCV RNA < assay at treatment week W_12_ = P_eRVR_
4. Probability of attaining HCV RNA < assay 24 weeks after therapy end = P_SVR_
5. Probability of withdrawing from therapy for any cause = P_withdrawl_
6. Proportion of all therapy withdraws that are attributable to major treatment toxicity = P_tox_

The user can define an algorithmic response to events such as virologic failure, attaining both 4 and 12 week HCV RNA < assay (extended rapid virologic response), or having HCV RNA detectable, but less than 1,000 copies/ml. For example, when modeling response guided therapy using pegylated interferon, ribavirin, and an HCV protease inhibitor, having an HCV RNA > 1,000 copies/ml leads to treatment cessation, having detectable HCV RNA that is < 1,000 copies/ml at either treatment week 4 or 12 leads to the course of therapy being extended from 24 to 48 weeks. Having undetectable HCV RNA at both weeks 4 and 12 leads to a 24-week total treatment course.

In every month while on therapy, individuals face a probability of withdrawal for toxicity, inability to tolerate adverse effects of interferon, or non-adherence. Among those who withdraw for any reason, the model determines whether that withdrawl from therapy was the result of treatment-ending toxicity. Those who experience treatment-ending toxicity have a one-month decrement in quality of life related to the toxicity, as well as a one-time cost associated with managing the toxicity. Individuals who stop treatment due to toxicity accrue a cost that reflects the cost of an “average” toxicity event. This cost was calculated by identifying the most common toxicity event types, estimating the cost of managing each type of toxicity event, and taking the weighted average cost of all toxicity event types using the proportion of toxicity events attributable to each toxicity type as the weight (Table S1).

In addition to treatment-ending toxicity, a portion of individuals on HCV therapy during any month experience non-treatment ending toxicity related to conditions such as rash, anemia, neutropenia, or other causes such as depression. To calculate non-treatment ending toxicity costs, we took the same approach as for treatment-ending toxicities and assigned these costs on a monthly basis. This “average” toxicity cost is included in the cost of HCV therapy for all patients who start treatment.

Non-HCV morbidity and mortality

In addition to the HCV-attributable causes of death, in every month individuals in the model are exposed to risk of death from causes other than HCV, as well as costs associated with non-HCV healthcare. Competing risk mortality is age- and sex-stratified, and is informed by U.S. vital statistics data [[1](#_ENREF_1)]. In addition, competing risk mortality is adjusted using standardized mortality ratios, to reflect elevated competing risks of death in persons with a history of HCV risk-behaviors such as intravenous drug use [[2](#_ENREF_2)].

Costs

The model generates cost estimates from 2 perspectives:

1. Health system perspective: these costs include those of all HCV-related care, including hospitalizations, emergency department visits, clinic visits, and medications, as well as healthcare-associated costs not related to HCV. Non-HCV costs are a function of age and sex [[3](#_ENREF_3),[4](#_ENREF_4)].
2. Program costs: these costs are tabulated from the perspective of a provider, such as a social service agency or medical home, that has been funded by a health department or other payer to improve follow-up along the HCV cascade of care. These costs only include direct and indirect costs related to ongoing program administration. For example, program costs include labor, benefits, and overhead for intervention staff, but do not include the cost of HCV medications, hospital visits, or the costs of program start-up.

In every month that an individual in the model receives intervention services, the model tabulates an individual monthly intervention cost. Typically, individual monthly costs are estimated by calculating the annual cost for full intervention staff and supplies, dividing that annual cost by the caseload of intervention workers to find the individual annual cost, and then dividing the individual annual cost by 12 to find the individual monthly cost. If an individual is lost to follow-up during the course of the intervention, the stream of program costs is disrupted, such that only individuals who complete the entire intervention course accrue the full cost. The individual who is lost to follow-up is assumed to be replaced by a new individual in the following month such that the average worker caseload remains unchanged.

**REFERENCES**

1. Centers for Disease Control and Prevention (2008) United States Life Tables. Available: <http://www.cdc.gov/nchs/products/life_tables.htm>. Accessed 3 May 2012.

2. Arendt M, Munk-Jorgensen P, Sher L, Jensen SO (2011) Mortality among individuals with cannabis, cocaine, amphetamine, MDMA, and opioid use disorders: a nationwide follow-up study of Danish substance users in treatment. Drug Alcohol Depend 114: 134-139.

3. Vastag B (2003) In-office opiate treatment "not a panacea": physicians slow to embrace therapeutic option. JAMA 290: 731-735.

4. Davis KL, Mitra D, Medjedovic J, Beam C, Rustgi V (2011) Direct economic burden of chronic hepatitis C virus in a United States managed care population. J Clin Gastroenterol 45: e17-24.

5. Micromedex 2.0 (2011) Drug Topics Red Book Online. Available: <http://www.micromedexsolutions.com>. Accessed 1 Februrary 2013.

6. Gao X, Stephens JM, Carter JA, Haider S, Rustgi VK (2012) Impact of adverse events on costs and quality of life in protease inhibitor-based combination therapy for hepatitis C. Expert Rev Pharmacoecon Outcomes Res 12: 335-343.

7. Kakko J, Svanborg KD, Kreek MJ, Heilig M (2003) 1-year retention and social function after buprenorphine-assisted relapse prevention treatment for heroin dependence in Sweden: a randomised, placebo-controlled trial. Lancet 361: 662-668.

8. Whitaker B, Sullivan M (2005) The 2005 Nationwide Blood Collection and Utilization Survey Report. Washingon, D.C.: Department of Health and Human Services.

9. Sohler NL, Wong MD, Cunningham WE, Cabral H, Drainoni ML, et al. (2007) Type and pattern of illicit drug use and access to health care services for HIV-infected people. Aids Patient Care and STDS 21 Suppl 1: S68-76.

10. McHutchison JG, Lawitz EJ, Shiffman ML, Muir AJ, Galler GW, et al. (2009) Peginterferon alfa-2b or alfa-2a with ribavirin for treatment of hepatitis C infection. N Engl J Med 361: 580-593.

11. Physician's Desk Reference (2011) Available: <http://www.pdr.net>. Accessed 15 May 2012.

12. Food and Drug Administration (2012) Telaprevir package insert. Available: [www.fda.gov](http://www.fda.gov). Accessed 13 March 2013.

13. Levinson DR (2005) Medicaid drug price comparisons: average manufacturer price to published prices.

14. United States Department of Health and Human Services Center for Medicare Services (2011) Physician Fee Schedule. Available: <http://www.cms.gov/Medicare/Medicare-Fee-for-Service-Payment/PhysicianFeeSched/index.html?redirect=/PhysicianFeeSched>. Accessed 15 January 2013.

15. United States Department of Labor (2011) National Compensation Survey. Available: [www.bls.gov/data](http://www.bls.gov/data). Accessed 10 February 2012.

16. Hawthorne G, Richardson J, Osborne R (1999) The Assessment of Quality of Life (AQoL) instrument: a psychometric measure of health-related quality of life. Qual Life Res 8: 209-224.

17. Centers for Disease Control and Prevention ARTAS Linkage Case Management Implementation Manual. Washington, D.C.: Academy for Edicational Development Center on AIDS and Community Health.

18. New York City Department of Health and Mental Hygiene (2012) Request for Proposals: Check Hep C: Demonstration Project for Providing Comprehensive Hepatitis C Community-Based Services for New Yorkers with Hepatitis C

19. Gardner LI, Metsch LR, Anderson-Mahoney P, Loughlin AM, del Rio C, et al. (2005) Efficacy of a brief case management intervention to link recently diagnosed HIV-infected persons to care. AIDS 19: 423-431.

20. United States Institutes of Health (2010) Rapid HIV Testing and Counseling NCT00809445. Available: <http://clinicaltrials.gov/ct2/show/NCT00809445?term=HIV+Rapid+Testing+and+Counseling&rank=1>. Accessed 5 November 2012.

21. Internal Revenue Service (2011) Mileage Rate: 55.5 Cents per Mile. Available: <http://www.irs.gov/uac/IRS-Increases-Mileage-Rate-to-55.5-Cents-per-Mile>. Accessed 3 May 2012.

**Supplemental tables and figures**

**Figure S1. HEP-CE model flow diagram**

**Figure S1**

Yes

Probability of developing incident HCV in the future

Does the individual enter the model HCV-infected (prevalent HCV)?

Individual enters HEP-CE model

Random numbers assign unique values for:

- Age
- Sex
- HCV genotype
- Age of infection
- Time to cirrhosis

HCV un-infected 🡻

Is the individual screened for HCV?

HCV disease progression 🡻

Probability rescreened in the future

Does the individual go through HCV cascade of care and initiate treatment?

Probability of re-engaging in HCV care in the future

Does the individual complete therapy?

HCV un-infected 🡻

Probability of re-infection with incident HCV in the future

No

Yes

Yes

No

No

Yes

Does the individual achieve SVR?

No

Yes

Unaware of HCV infection/HCV disease progression 🡻

HCV disease progression/ no chance of re-treatment 🡻

**Key**

🡻 Chance of death from competing mortality

Individual enters model for this analysis

| Table S1. Treatment-ending Toxicity Costs (2011 U.S. $) | | | | | |
| --- | --- | --- | --- | --- | --- |
| Resource Utilized | CPT code | Unit Cost | # Utilized | Total Cost | Source |
| Anemia |  |  |  |  |  |
| Epotein alfa | N/A | 485.10 | 1 | 485.10 | [[5](#_ENREF_5),[6](#_ENREF_6)], Expert opinion |
| Complete blood count | 85,025 | 10.94 | 3 | 32.82 | [[6](#_ENREF_6),[7](#_ENREF_7)], Expert opinion |
| Bilirubin | 82,247 | 7.06 | 1 | 7.06 | [[6](#_ENREF_6),[7](#_ENREF_7)], Expert opinion |
| Uric acid | 84,550 | 6.36 | 1 | 6.36 | [[6](#_ENREF_6),[7](#_ENREF_7)], Expert opinion |
| Reticulocytes | 85,045 | 5.63 | 3 | 16.89 | [[6](#_ENREF_6),[7](#_ENREF_7)], Expert opinion |
| Transfusion | 96,365 | 54.37 | 1 | 54.37 | [[6](#_ENREF_6),[7](#_ENREF_7)], Expert opinion |
| Packed red blood cells | P9021 | 231.50 | 2 | 463.00 | [[6](#_ENREF_6),[8](#_ENREF_8)], Expert opinion |
| Clinic visit and injection | 99,211 | 19.74 | 2 | 39.48 | [[6](#_ENREF_6),[9](#_ENREF_9)], Expert opinion |
| Moderately complex clinic visit | 99,214 | 104.16 | 2 | 208.32 | [[9](#_ENREF_9)], Expert opinion |
| Proportion with anemia | __ | __ | __ | 0.097 | [[10](#_ENREF_10)] |
| *Total cost of anemia* | __ | __ | __ | *$1,313.40* |  |
| Rash |  |  |  |  |  |
| Clobetasol propionate cream (150 gm) | N/A | 35.86 | 1 | 35.86 | [[5](#_ENREF_5),[6](#_ENREF_6)], Expert opinion |
| Simple clinic visit | 99,213 | 70.46 | 2 | 140.92 | [[6](#_ENREF_6),[9](#_ENREF_9)], Expert opinion |
| Dermatology consultation | 99,243 | 119.81 | 1 | 119.81 | [[6](#_ENREF_6),[9](#_ENREF_9)], Expert opinion |
| Proportion with rash | __ | __ | __ | 0.387 | [[10](#_ENREF_10)] |
| *Total cost of rash* | *__* | *__* | *__* | *$269.59* |  |
| Other SAE |  |  |  |  |  |
| Moderately complex clinic visit | 99,214 | 104.16 | 2 | 208.32 | [[9](#_ENREF_9)], Expert opinion |
| Complete blood count | 85,025 | 10.94 | 1 | 10.94 | [[7](#_ENREF_7)], Expert opinion |
| Liver function test | 80,069 | 12.22 | 1 | 12.22 | [[7](#_ENREF_7)], Expert opinion |
| Proportion with other SAE | __ | __ | __ | 0.516 | [[10](#_ENREF_10)] |
| *Total cost of other SAE* | *__* | *__* | *__* | *$231.48* |  |
| Total cost of treatment ending toxicity | | |  | **$361.36^a^** |  |

CPT = current procedural terminology; SAE= serious adverse event

^a^ Total costs equal weighted average of toxicity costs:

(Proportion with anemia * anemia costs + proportion with rash * rash costs + proportion with other SAE * SAE costs)

**Table S2. HCV therapy costs (2011 U.S. $)**

| Drug | HCV genotype | Standard dose | Frequency | Cost per month | Reference |
| --- | --- | --- | --- | --- | --- |
| Ribavirin |  |  |  |  |  |
| Standard HCV treatment ^a,b^ | 1 | 600 mg | 2/day | $1,371 | [[11](#_ENREF_11),[12](#_ENREF_12)] |
| Standard HCV treatment ^c^ | 2,3 | 400 mg | 2/day | $914 | [[11](#_ENREF_11)] |
| Anemia (dose reduction) ^c^ | All | 600 mg | 1/day | $685 | [[11](#_ENREF_11)] |
| Pegylated interferon alfa-2a | | | | | |
| Standard HCV treatment ^d^ | All | 180 mcg/ml | 1/week | $2,097 | [[11](#_ENREF_11),[12](#_ENREF_12)] |
| Neutropenia (dose reduction) | All | 135 mcg/ml | 1/week | $1,572 | [[11](#_ENREF_11)] |
| Telaprevir | | | | | |
| Standard HCV treatment ^e^ | 1 | 750 mg | 3/day | $15,154 | [[12](#_ENREF_12)] |
| Filgrastim | | | | | |
| Neutropenia ^d^ | All | 300 mcg/ml | 2/week | $1,900 | [[11](#_ENREF_11)], Expert opinion |
| Clobetasol propionate | | | | | |
| Rash | 1 | 150 gm | 1/month | $160 | [[11](#_ENREF_11),[12](#_ENREF_12)], Expert opinion |

Note: the monthly cost estimate is based on the average wholesale price [[5](#_ENREF_5)], less 23% [[13](#_ENREF_13)], assuming a standard dosage and number of pills for 1 month. 12.0% [[10](#_ENREF_10)] incur an additional cost of 19.75 [[14](#_ENREF_14)] for a nurse visit to treat an adverse event. Ribavirin doses assume an average cohort weight of 80 kg or less.Treatment of neutropenia includes peginterferon alfa-2a dose reduction and use of filgrastim.

^a^ 18.0% receive ribavirin dose reduction to 600 mg/day to treat anemia (months 1-3 *only*)

^b^ 8.5% receive ribavirin dose reduction to 600 mg/day to treat anemia (months 4-12 *only*)

^c^ 8.5% receive ribavirin dose reduction to 600 mg/day to treat anemia (all months)

^d^ 6.5% receive peginterferon alfa-2a dose reduction from 180 to 135 mcg/ml to treat neutropenia and 300 mg of filgrastim twice per week (all months)

**Table S3. Intervention Cost Inputs (2011 U.S. $)**

| Cost Inputs | Value | Source |
| --- | --- | --- |
| Hourly wage rates | | |
| Case manager ^a,b^ | $25.56 | [[15](#_ENREF_15)] |
| Contracts manager | $38.39 | [[15](#_ENREF_15)] |
| Evaluator | $36.08 | [[15](#_ENREF_15)] |
| Peer navigator ^c^ | $25.26 | [[15](#_ENREF_15)] |
| Program director | $31.64 | [[15](#_ENREF_15)] |
| Registered nurse ^a^ | $33.79 | [[15](#_ENREF_15)] |
| Program supervisor | $43.95 | [[15](#_ENREF_15)] |
| Physician ^a^ | $97.23 | [[15](#_ENREF_15)] |
| Fringe benefits, overhead, and other | | |
| Fringe | 44.0% | [[15](#_ENREF_15)] |
| Overhead ^d^ | 45.4% | [[16](#_ENREF_16)] |
| Work hours per week | 40 | Assumption |
| Work weeks per year | 50 | Assumption |
| Time estimate | | |
| Case manager (FTE) ^a,b^ | 1 | [[17](#_ENREF_17)] |
| Contracts manager (FTE) | 0.25 | [[17](#_ENREF_17)] |
| Evaluator (FTE) | 0.15 | [[17](#_ENREF_17)] |
| Peer navigator (FTE) ^c^ | 1 | [[18](#_ENREF_18)] |
| Program director (FTE) |  |  |
| Linkage and peer navigator interventions | 0.325 | [[17](#_ENREF_17)] |
| Treatment initiation | 0.25 | [[17](#_ENREF_17)] |
| Program supervisor (FTE) | 0.07 | [[17](#_ENREF_17)] |
| Registered nurse (minutes) ^a,e^ | 30 | Assumption |
| Physician (minutes) ^a,f^ | 25 | [[9](#_ENREF_9)] |
| Intervention duration (months) ^g^ | | |
| Linkage | 3 | [[19](#_ENREF_19)] |
| Treatment initiation | 3 | Assumption |
| Integrated case management | 6 | Assumption |
| Peer navigator | 12-18 | [[18](#_ENREF_18)], Assumption |
| Average yearly case load | | |
| Low ^h^ | 50 | [[18](#_ENREF_18)] |
| Mid ^i^ | 120 | [[19](#_ENREF_19)] |
| High ^j^ | 250 | [[18](#_ENREF_18)] |
| Travel ^c^ | | |
| Number of round trips | 20 | Assumption |
| % taking public transportation ^g^ | 26.8% | [[20](#_ENREF_20)] |
| Cost per round trip |  |  |
| Public transportation | $2.80 | [[20](#_ENREF_20)] |
| Private car |  |  |
| Number of miles traveled per round trip | 25 | Assumption |
| Cost per mile | $0.53 | [[21](#_ENREF_21)] |
| Other | | |
| Materials (cost per month) | $0.50 | Assumption |

FTE: full-time equivalent; BLS = Bureau of Labor Statistics

Note: average BLS wage rates are from the Ambulatory Health Care Services industry.

^a^ Used in treatment initiation intervention *only*

^b^ Used in linkage intervention *only*

^c^ Used in peer navigator intervention *only*

^d^ Applied to wage rate and fringe

^e^ Two visits

^f^ One visit

^g^ Assumed those not taking public transportation drove private car

^h^ Best estimate for peer navigator intervention

^i^ Best estimate for linkage intervention

^j^ Best estimate for treatment initiation intervention

**Table S4. Interventions cost per patient (2011 U.S. $)**

| Cost Component | Linkage | | Treatment Initiation | | Integrated Case Management ^a^ | | Peer Navigator | |
| --- | --- | --- | --- | --- | --- | --- | --- | --- |
|  | **Base case** | **Range** | **Base case** | **Range** | **Base case** | **Range** | **Base case** | **Range** |
| Labor |  |  |  |  |  |  |  |  |
| Clinical | 613 | (294-1,472) | 401 | (401-1,579) | 1,015 | (696-3,052) | 1,892 | (378-1,892) |
| Supervisor/other | 681 | (327-1,634) | 299 | (299-1,497) | 490 | (313-1,566) | 1,634 | (327-1,634) |
| Overheard | 588 | (282-1,410) | 318 | (318-1,397) | 683 | (458-2,096) | 1,601 | (320-1,601) |
| Materials | 2 | (2-2) | 2 | (2-2) | 3 | (3-3) | 8 | (8-8) |
| Travel | --- | --- | --- | --- | --- | --- | 210 | (210-210) |
| Total cost per patient ^b^ | **1,883** | **(905-4,518)** | **1,021** | **(1,021-4,475)** | **2,191** | **(1,470-6,716)** | **5,344** | **(1,243-5,344)** |

^a^ The integrated case management program is comprised of two separate linkage and treatment initiation interventions. As such, the program costs listed here are an *average* of the different cost assumptions of the linkage and treatment initiation interventions by themselves with some economies of scale and cost savings of combining two interventions into one overall program.

^b^ These total costs assume the patient does not drop out during the intervention.
